# S1 guideline sweat gland carcinoma

**DOI:** 10.1111/ddg.70107x

**Published:** 2026-07-07

**Authors:** Mirjana Ziemer, Michael Erdmann, Abbas Agaimy, Jürgen C. Becker, Almut Böer‐Auer, Doris Helbig, Cornelia Erfurt‐Berge, Alexander Frisman, Conrad Hempel, Ulrike Leiter, Friedegund Meier, Thomas Mentzel, Lara Racz, Silke Redler, Stefan Schliep, Clemens Seidel, Alpaslan Tasdogan, Jochen Utikal, Carsten Weishaupt, Selma Ugurel

**Affiliations:** ^1^ Department of Dermatology Venereology and Allergology Skin Tumor Center Leipzig Cancer Center Central Germany (CCCG) University Hospital Leipzig Leipzig Germany; ^2^ Department of Dermatology University Hospital Erlangen Comprehensive Cancer Center (CCC) Erlangen‐EMN Bavarian Center for Cancer Research (BZKF) Friedrich‐Alexander University of Erlangen‐Nuremberg Erlangen Germany; ^3^ Institute for Pathologie University Hospital Erlangen Friedrich‐Alexander University of Erlangen‐Nuremberg Erlangen Germany; ^4^ Translational Skin Cancer Research German Cancer Consortium (DKTK) University Medical Center Essen Germany German Cancer Research Center (DKFZ) Heidelberg Germany; ^5^ Dermatologikum Hamburg GmbH Hamburg Germany; ^6^ Department of Dermatology and Venereology University Hospital Cologne Cologne Germany; ^7^ Department of Radiation Therapy University Hospital Leipzig Leipzig Germany; ^8^ Department of Dermatology University Hospital Tübingen Tübingen Germany; ^9^ Department of Dermatology National Center for Tumor Diseases/University Cancer Center Dresden Dresden Germany; ^10^ Kressbronn, Germany; ^11^ Institute for Human Genetics Medical Faculty and University Hospital Düsseldorf Heinrich Heine University Düsseldorf Düsseldorf Germany; ^12^ Department of Dermatology Venereology and Allergology University Medical Center Essen Essen Germany; ^13^ Clinical Cooperation Unit Dermato‐Oncology of the German Cancer Research Center Heidelberg (DKFZ) and the Department of Dermatology Venereology and Allergology University Medical Center Mannheim Ruprecht Karl University of Heidelberg Mannheim Germany and DKFZ Hector Cancer Institute at the University Medical Center Mannheim Mannheim Germany; ^14^ Department of Dermatology and Venereology University Hospital Münster Münster Germany; ^15^ Department of Dermatology Venereology and Allergology Bielefeld Rosenhöhe Clinic Campus Medical Faculty and University Hospital OWL Bielefeld University Bielefeld Germany

## Abstract

The current classification of sweat gland carcinomas is based on histomorphological characteristics and distinguishes between more than 20 entities. Most patients are older, but some subtypes also affect middle‐aged and younger patients. The majority of tumors arise *de novo*. Sweat gland carcinomas have nonspecific clinical features. The tumors are usually located in the head and neck area and on extremities, with the exception of extramammary Paget's carcinoma, which has an anogenital predilection. Sweat gland carcinomas occurring in the armpit may pose a histomorphological challenge in distinguishing them from metastatic or primary breast cancer. The diagnosis is made histopathologically via excisional biopsy. Histopathological subdifferentiation is essential for an accurate diagnosis. Metastasis initially occurs locally (per continuitatem), later to regional lymph nodes and distant organs. The treatment of choice is complete surgical excision with entire histopathological margin control (microscopically controlled surgery) or alternatively with wide local excision with a safety margin. Postoperative adjuvant radiotherapy is recommended for high‐risk tumors. The data available for drug therapy of advanced tumors is generally weak, with the best results seen for chemotherapy using platinum derivatives. Furthermore, targeted therapies are possible, e.g., with HER2/neu or EGFR inhibitors, possibly in combination with chemotherapy. A risk‐adapted regimen is recommended for the follow‐up care.

## EPIDEMIOLOGY AND ETIOPATHOGENESIS

*Chapter authors*: Conrad Hempel, Mirjana Ziemer, Michael Erdmann

### Epidemiology

Sweat gland carcinomas are rare neoplasms of the skin.^1^ They represent a group of various entities with the common feature of originating from apocrine or eccrine sweat glands of the skin. Overall, sweat gland carcinomas account for only approximately 0.05 % of all malignant skin tumors.^2^ However, they represent the major fraction within the adnexal carcinomas (incidence 5.1 per 10^6^) – after basal cell carcinoma as tumor with follicular differentiation.^1^, ^3^ Their incidence is increasing.^2^, ^4^ When considering all entities, the proportions of affected men and women are not significantly different. A special feature of some entities – for example, digital papillary adenocarcinoma – is the young age of the patients. The topography of the tumors is also important, given that tumors with highly similar morphology, but different biology, occur in organs close to the skin, such as salivary glands as well as the breast.

Reliable differentiation of the individual sweat gland tumor entities is of prognostic relevance. While some entities, such as microcystic adnexal carcinoma, have a very good prognosis after verified complete excision, other entities have poorer survival rates and need, therefore, respective differentiated treatment and follow‐up.

### Etiology

Most adnexal carcinomas arise *de novo* and sporadically. Malignant transformation of benign tumors is less common.^3^ Potential etiopathogenetic trigger factors may include UV radiation, preceding radiotherapy, existing immunosuppression, but also causative genetic diseases.^5^ Especially with respect to porocarcinoma and hidradenocarcinoma, there is growing evidence suggesting a role of oncogenic drivers in PI3K/AKT (phosphoinositide 3‐kinase/protein kinase B) and MAPK (mitogen‐activated protein kinase) signaling pathways in tumorigenesis. In addition, a high tumor mutational burden attributed to UV exposure has been identified in porocarcinomas.^6^, ^7^ HPV‐associated tumor development has been demonstrated for digital papillary adenocarcinoma.^8^ Specific genetic alterations have been detected in some tumors, for example, *MYB::MYBL1* fusion in adenoid cystic carcinoma and*YAP1::NUTM1, YAP1::MAML2*, or *YAP1::WWTR1* fusions in porocarcinoma. Brooke‐Spiegler syndrome is a tumor‐associated genetic disease with increased incidence of sweat gland carcinomas.^9^

## CLASSIFICATION

*Chapter authors*: Mirjana Ziemer, Almut Böer‐Auer

Histologically, sweat gland carcinomas mimic ductal structures and/or secretory end pieces of apocrine or eccrine glands.

Apocrine glands (*glandulae sudoriferae apocrinae*) are part of the embryonic apocrine‐follicular‐sebaceous unit and open above the sebaceous glands into the hair follicle (occasionally also directly to the epidermis). Their distribution pattern is easier to remember by the fact that they are predominantly localized is skin regions starting with the letter “a”: armpits (axillary), nipples (areolar), anogenital, face (German: *Antlitz*), especially eyelids (German: *Augenlider*). Occasionally, they are also found in pubic and periumbilical areas. Ceruminous glands of the external auditory canal and Moll's glands on the margins of the eyelids are considered modified apocrine glands.

Eccrine glands (*glandulae sudoriferae eccrinae*) are independent of the hair follicle and open via an acrosyringium directly to the epidermis.

Classification of tumors in eccrine and apocrine differentiation is, however, not always possible, given that ductal structures of both glands have an identical structure while differentiating features, such as apocrine secretion or association with embryologically related tissues like hair follicle or sebaceous gland differentiation are often lacking. Moreover, both apocrine and eccrine variants have been described for many entities. Ultimately, however, the differentiation has currently no therapeutic consequences.

First classifications of sweat gland carcinomas from the 1950s were primarily based on particular histomorphological characteristics. For example, Stout and Cooley divided the tumors into either adenoid cystic, mucinous, or ductal carcinomas.^10^ In 1968, Berg and McDevitt presented an advanced classification based on a series including more than 100 cases.^11^

Another possibility is the differentiation in low‐malignant and high‐malignant tumors based on the risk of developing metastatic disease. While low‐risk tumors may have a higher risk of recurrence if surgery is performed without safety margin or is not microscopically controlled, they rarely form metastases. When they do, it is usually to locoregional lymph nodes.

Despite their rarity, sweat gland tumors, in particular, have developed a multitude of subtypes that are, in part, difficult to distinguish from each other. This applies to both benign sweat gland tumors and their malignant counterparts. As yet, no systematic classification exists – apart from the histopathological WHO classification –, which makes it difficult to provide a clear overall assessment of the group. More than 20 malignant entities have been described and the most important of these are presented in Table 1.^12^ In many cases, the subtypes of sweat gland carcinomas are variants of their benign counterparts. Another difficulty is that morphologically very similar tumors either with the same or with divergent terminology but with sometimes a very different prognosis may occur in other organs close to the skin, such as salivary glands as well as the breast. Depending on the topography, it must, therefore, first be determined whether the tumor actually is an adnexal tumor originating from the skin or whether other organs must be considered as the site of origin. This analysis may be supported by immunophenotyping.

The codes of sweat gland carcinomas in ICD‐O comprise the numbers 839–842 in the chapter *Tumors of skin and skin appendages* (for example, 8400/3 sweat gland carcinoma).

## DIAGNOSIS

*Chapter authors*: Almut Böer‐Auer, Mirjana Ziemer, Thomas Mentzel, Abbas Agaimy, Doris Helbig, Stefan Schliep

### Clinical appearance

With a few exceptions, sweat gland carcinomas arise in the head and neck area usually at an advanced age.^1^ They have no specific clinical features and manifest frequently in the form of erythematous, livid nodules and nodes. In contrast to their benign counterparts, sweat gland carcinomas are often prone to ulceration. Tentative diagnoses include benign cysts, basal cell carcinomas, or cutaneous squamous cell carcinomas. Extramammary Paget's carcinoma and signet ring cell carcinoma of the skin are exceptions initially often misdiagnosed as inflammatory dermatosis. Patients with sweat gland carcinomas are usually asymptomatic.^13^ In clinical examination and history, attention should be paid to risk factors, such as UV radiation, preceding radiotherapy, immunosuppression, HPV infection, genetic aberrations, and genetic syndromes, such as Brooke‐Spiegler syndrome, as well as hematoproliferative disorders (in particular, chronic lymphocytic leukemia).

### General histopathology

Due to their common features, reliable histomorphological classification of sweat gland carcinomas in apocrine and eccrine differentiation is not always possible. Excretory ducts of eccrine and apocrine glands have an identical structure. The stratified ductal epithelium consists of two layers of small eosinophilic cells forming an eosinophilic cuticle towards the lumen. The peripheral cells are more basophilic with a cuboid shape and round nuclei. A myoepithelial outer cell layer is absent. The secretory end pieces consist of one layer of cuboid or columnar cells surrounded by myoepithelial cells.

Criteria for tumors with apocrine differentiation include:

− evidence of so‐called “decapitation secretion” (cytoplasmic portions close to the lumen are pinched off and released as secretions into the lumen of the gland),
− elongated and branched tubular structures,
− connection of the tumor to an infundibulum of a hair follicle,
− tumor portions with features of follicular and/or sebaceous differentiation (especially in benign adnexal tumors or sweat gland carcinomas developing in their benign counterpart),
− special cytological features: abundant granular eosinophilic cytoplasm with isomorphic central nuclei and apparent nucleoli.

In contrast, eccrine secretory differentiation is characterized by:

− flat secretory epithelium without morphological secretion into the lumen.

Both apocrine and eccrine secretory end pieces have a peripheral myoepithelial layer, so that sweat gland tumors may exhibit a prominent myoepithelial differentiation. Given that evidence of such a biphasic (epithelial and myoepithelial) structure may also be present in carcinomas, it cannot be used as general marker for benign tumors.

In contrast to their benign counterparts (if present), the histopathology of sweat gland carcinomas is, in general, characterized by cell atypia, nuclear pleomorphism, closely packed and overlapping nuclei (crowding), numerous mitoses, cell necrosis, and infiltrative tumor growth.

It should be noted that histopathology is not in all cases suitable as predictor for the clinical course, given that a part of the tumors may show aggressive behavior despite an inconspicuous histopathological pattern.

#### Immunophenotyping and molecular‐pathological markers

Immunohistochemically, the glandular epithelium of both apocrine and eccrine glands can be visualized (apart from pan‐cytokeratin markers) with cytokeratin (CK) 7, CK8, CK18, and *gross cystic disease fluid protein 15* (GCDFP‐15, which is otherwise a typical marker for breast cancer). Cells close to the glandular lumen are stained with antibodies against CEA (carcinoembryonic antigen) and EMA (epithelial membrane antigen). These stainings are, however, not specific. The secretory end pieces of apocrine and eccrine glands are surrounded by an outer layer of flat myoepithelial cells that can be visualized by the markers smooth muscle actin, p63, or calponin. In all digital papillary adenocarcinomas, for example, the myoepithelial pattern can be visualized by p63 in immunohistochemistry, whereas p63 staining shows a diffuse pattern in porocarcinoma and clear cell hidradenocarcinoma. However, the expression patterns may be less consistent in sweat gland carcinomas than in their benign counterparts or in regular glands. In this context, metaplastic phenomena like squamoid or mucinous metaplasia should also be considered.^15^

Sweat gland carcinomas frequently express EGF receptor (epidermal growth factor receptor, EGF‐R). In differential diagnosis, this may be useful for distinguishing them from metastases of breast cancer, given that EGF‐R expression in breast cancer is markedly less common. However, its insufficient specificity has to be considered, especially concerning the distinction from other tumors with squamous differentiation. In contrast, there is no reliable difference between sweat gland carcinomas and breast cancer metastases with respect to the expression of estrogen and/or progesterone receptors.^14^ Especially in mucinous sweat gland carcinomas the profile is identical to mucinous breast cancer with strong homogeneous expression of estrogen and progesterone receptors and often also of synaptophysin. Identification of estrogen and/or progesterone receptors may play a role for a potential antihormone therapy with tamoxifen.^15^ Global expression of androgen receptors is also a regular feature of apocrine adenocarcinomas. Moreover, expression of CK5/6 and p63 indicate a primary sweat gland carcinoma rather than metastasis.

In recent years, additional immunohistochemical and molecular markers have been established that may facilitate differentiation of sweat gland carcinomas. For example, all porocarcinomas show either activation of NUT (nuclear protein of testis) by positive immunohistochemical staining or a *YAP1::NUTM1* fusion gene in RNA sequencing (yes‐associated protein [YAP] 1). *MAML2* fluorescence in situ hybridization (FISH) is positive in all clear cell hidradenocarcinomas.^15^

Of interest, especially with respect to the prognosis but also the therapeutic approach, are PD‐L1 expression and the fraction of tumor‐infiltrating CD8‐positive lymphocytes.^16^ These were analyzed by immunohistochemistry on 83 adnexal carcinomas including 60 sweat gland carcinomas. Of these, 13 % expressed PD‐L1 in ≥ 1 % of the tumor cells (8/60). Apocrine carcinomas (40 %, 2/5) and invasive extramammary Paget's carcinomas (57 %, 4/7) showed the highest PD‐L1 expression on tumor cells. Immune cells expressed PD‐L1 significantly more often than tumor cells. Dense CD8‐positive tumor‐infiltrating lymphocytes were found in 43 % of sweat gland carcinomas. While the expression of PD‐L1 by tumor cells was associated with metastasis formation in the univariate analysis (hazard ratio [HR] 4.0, 95 % confidence interval [CI] 1.1–15, p = 0.04), this was not the case in the multivariate analysis (HR 4.1, 95 % CI 0.6–29, p = 0.15).

In sweat gland carcinomas developing in their benign counterparts, expression of the proliferation marker Ki‐67 is enhanced in foci of the malignant portion. Otherwise, the proliferation marker is very variable und not always significantly enhanced, especially in highly differentiated sweat gland carcinomas.

### Specific clinical features and histology of sweat gland carcinomas

In the following, the most common clinical variants are addressed in more detail. The most important characteristics of additional sweat gland carcinomas are presented in Table 1.

**TABLE 1:** Clinical, histopathological, and molecular pathological characteristics as well as differential diagnostic features of sweat gland carcinomas.

	Clinical appearance	Age	Localization	Histological characteristics	Immunohistochemical features	Molecular pathology Mutations and copy number variations	Aspects of differential diagnosis
***Subtypes of sweat gland carcinomas with benign counterparts***							
***Porocarcinoma*** (*Syn*.: malignant poroma, epidermotropic eccrine carcinoma, malignant hidroacanthoma simplex)	Reminiscent of squamous cell carcinoma or basal cell carcinoma	Predominantly older patients	44 % lower extremities, 24 % trunk, 24 % head and neck	Atypical poroid cells, focally ductal differentiation, numerous mitoses, extensive tumor necrosis possible, clear cell and squamoid differentiations quite common pigmentation due to colonization with melanocytes intralymphatic spread in tumor periphery	*Positive*: CK7, NUT	Approximately 280 reported tumors: Generally high TMB, mainly traced back to signatures of mutational processes in association with UV radiation *YAP1* fusion genes *PAK1* fusion genes *PAK2* fusion genes *PAK3* fusion genes Other fusion genes: 1 x *WWTR1::NUTM1*, 1 x *PIK3R1::YTHDC2* 8 x 1.6 Mb amplification in 11q13.3 (comprising the genes *CCND1*, *FGF3*, *FGF4*, *FGF19*) 7 x 6.9 Mb deletion in 9p21.3 (comprising the genes *CDKN2A/B*, *MTAP*, *TEK*, *MOB3B*) 44 x *TP53* mutations 14 x *HRAS* mutations 11 x *KMT2D* mutations 11 x *LRP1B* mutations 9 x *CDKN2A* mutations 9 x *PIK3CA* mutations	Necrosis *en masse* also possible in poromas malignant hidroacanthoma simplex must be differentiated histologically from pagetoid Bowen's disease and clonal seborrheic keratosis
***Hidradenocarcinoma*** (*Syn*.: clear cell eccrine hidradenocarcinoma, malignant clear cell acrospiroma, malignant eccrine acrospiroma)	Asymptomatic indurated, erythematous nodes	Middle age	40 % occipital 29 % facial 30 % extremities	Dermal to subcutaneous nodular tumor cytological atypies, infiltrative tumor growth, and tumor necroses high number of mitoses clear cell, mucinous areas, and squamous metaplasia possible	*Positive*: CK7, MAML2 fusion	130 reported tumors: 12 x *CRTC1::MAML2* rearrangement 9 x *MAML2* rearrangement 9 x MSS 6 x high TMB 5 x low TMB 2 x potential unbalanced translocation / disruption of *CRTC1* 9 x *EGFR* disomy, 3 x polysomy, 6 x trisomy 2 x *Her2/neu* amplification	Carcinoma development sometimes focally in existing hidradenomas, complete processing recommended atypical hidradenomas (especially “atypical clear cell hidradenomas” with intralymphatic tumor cells) should be excised *in toto* given the difficult histopathological differentiation from early hidradenocarcinoma in case of axillary tumors with histomorphology of hidradenocarcinoma consider metastasis/extension of primary breast cancer
***Malignant mixed tumor of the skin*** (*Syn*.: malignant apocrine mixed tumor of the skin, malignant chondroid syringoma of the skin) ***Myoepithelial carcinoma of the skin*** (*Syn*.: malignant cutaneous myoepithelioma)^68^	Subcutaneous nodes with possible ulceration and adhesion with the underlying tissue develop from existing mixed tumors or myoepitheliomas of the skin	Broad age spectrum, median age 50 Y	Mostly extremities (hands, feet), also face (myoepithelial carcinoma)	Parts still correspond to the existing mixed tumor, but mainly presenting with marked infiltrative growth, glandular and tubular structures still apparent, elongated, branched tubules and decapitation secretion possible in stroma, portions of hyaline, cartilaginous, and osseous structures predominantly, myoepithelial differentiation in myoepithelial carcinoma (ductal structures by definition absent)	*Positive*: PLAG1, less common HMGA2 in benign parts with apocrine differentiation^69^, ^70^, nuclear, variable intensity^71^ EMA, calponin, often also S100, CK, aSMA, sometimes GFAP^72^	No or no recurrent genetic alterations reported	Continuum from malignant mixed tumor to myoepithelial carcinoma analogous to the spectrum of benign counterparts if near salivary glands, important differential diagnosis of malignant mixed tumors of salivary glands (carcinoma derived from pleomorphic adenoma)
***Spiradenocarcinoma / cylindrocarcinoma*** ***spiradenocylindrocarcinoma*** ^73^ (all three represent the morphological ends of a common tumor spectrum)	Rapidly growing, ulcerating nodes, in existing spiradenomas / arising from multiple cylindromas in Brooke‐Spiegler syndrome	Usually > 50th year of life; median age 63 Y; in younger adulthood possible	Often extremities, also head and neck localization / mainly head and neck	Diagnosis only if portions of spiradenoma / cylindroma are still apparent often in deep corium or upper subcutis, infiltrative growth into subcutis possible loss of PAS‐positive basement membrane in cylindrocarcinoma increased cell density, marked cytological atypies, numerous mitoses, sometimes spindle cells often necrotic areas	Loss of PAS+ hyaline membranes positive: CK7, consistently p53 in malignant portions	63 reported spiradenocarcinomas: 6 x *TP53* mutations 5 x *TERT* mutations (hotspot *C228T*, *C250T*) signatures associated with DNA MMR high‐grade spiradenocarcinomas have large CNVs (for cylindrocarcinoma and spiradenocylindrocarcinoma no or no recurrent genetic alterations reported)	Hemorrhage and limited cytologic atypies and mitoses in spiradenomas with degenerative alterations, but no extensive tumor necroses
***Syringocystadenocarcinoma papilliferum*** ^74^	Erythematous nodes or plaques predominantly development from syringocystadenoma papilliferum	Mostly older patients, on average 6th decade of life	Often localization in head and neck area	Syringocystadenoma papilliferum still apparent infiltrative growth of tubular structures with cellular atypies tumor necrosis possible		15 reported tumors: 5 x *HRAS* mutations (of these, 4 x G13R) 5 x *TP53* mutations	
**Subtypes of sweat gland carcinomas without benign counterparts**							
***Extramammary Paget's carcinoma*** (*Syn*.: extramammary Paget's disease)	Poorly defined, partly erosive, erythematous plaque clinical features reminiscent of inflammatory dermatosis clinically inapparent extensions	Mean age at manifestation 70 Y	Mainly inguinal, perianal, perigenital, and axillary	Intraepithelial solitary or small groups of larger, bright cells with PAS‐positive cytoplasm potentially containing mucin ductal structures within the nests localization of the nests above a preserved keratinocytic row of basal cells poorly defined lateral margins spread along adnexal structures and into the deeper dermis	*Positive*: homogenous CK7 and CAM 5.2	> 500 reported tumors: up to 43 % amplifications and 15 % recurrent point mutations in *ERBB2* constitutive activation of PI3K‐AKT‐mTOR metabolism, often caused by mutations in *PI3KCA* (35 %, *p.E542K* variant), and *AKT1* several studies: *TOP2A* amplification in up to 40 % of cases several studies: mutations in p53 in approximately 30 % of cases – associated with advanced stage and poorer prognosis several studies: homozygous deletions of CDKN2A in 25 % of cases, associated with higher recurrence rates low TMB, rarely MSI (< 1 %)	Potential spread *per continuitatem* of colorectal and urogenital carcinomas in anogenital region; screening after underlying adenocarcinoma especially in perianal Paget's carcinoma (markedly less common in periscrotal and perivulval cases) CK7 and CAM 5.2 positivity allow for differentiation from pagetoid Bowen's disease and melanoma in situ (if necessary, also use of melanocytic markers) CK20 and CDX2 indicative of another underlying carcinoma
***Microcystic adnexal carcinoma*** (*Syn*.: sclerosing sweat gland carcinoma, eccrine epithelioma, syringomatous carcinoma)	Slowly growing, indurated plaque up to several centimeters in diameter	From the 3rd decade of life; frequently older patients	Head and neck (primarily cheeks), rarely trunk or extremities	Dermal to subcutaneous clusters of tumor cells without connection to epidermis in the upper portions, larger cystically dilated sweat gland excretion ducts similar to milia; in the deeper portions, small syringoid epithelial clusters or elongated tubular structures without conspicuous cytomorphological features or mitoses in hyaline, sclerosing, or mucinous stroma perineural spread in tumor periphery	*Positive*: CK7 negative: Ber‐EP4 (or only focally positive membranes)	25 reported tumors: 5 x *JAK/STAT* signaling pathway, calcium signaling pathway, *cGMP‐PKG* signaling pathway enriched 7 x CNVs in *JAK2*, *MAF*, *MAFB*, *JUN*, *FGFR1*	Differentiation from basal cell carcinomas with infiltrative growth, in particular infiltration of subcutis and perineural spread as most important evidence for differentiation from syringoma and desmoplastic trichoepithelioma, in particular absence of CK20‐positive Merkel cells for differentiation from desmoplastic trichoepithelioma differentiation from syringoid hamartomatous proliferations in familial syndromes typically affecting younger patients
***Digital papillary adenocarcinoma***	Slowly growing, solitary node	Adults, peak in 5th decade of life; men more often	Fingers, toes, rarely soles/palms	Dermal to subcutaneous, well‐defined tumor without connection to epidermis cystic or solid cystic structure predominantly basophil tumor cells with moderate cytological atypies; form often papillary structures; papillary structures may be absent; several mitoses squamous metaplasia and clear cell differentiation possible ultimately, broad morphological spectrum of hidradenoma‐ or cystadenoma‐like appearance through to highly pleomorphic infiltrative epithelial proliferations	Compartmentation of CK7‐positive epithelial and actin‐positive myoepithelial cells	Approximately 136 reported tumors: almost all HPV42‐positive 10 x *TP53* mutations 9 x *BRAF* mutations 7 x *EGFR* overexpression 6 x mutations in *KMT2B*, *MAML2*, *MAP3K1*, *CACNA1E*, *PDGFRA*, *HIF1A*, *PRDM16*, *TCF7L1* 4 x enrichment of genes associated with matrix remodeling, metabolism, DNA repair 2 x *CRTC3::MAM2* rearrangements 2 x *TRPS1::PLAG1* rearrangements	Missing compartmentation, e. g. in hidradenoma
***Adenoid cystic carcinoma***	Rough, reddish or skin‐colored node dermal and/or subcutaneous	Usually in the 6th decade of life; women slightly more often	Mainly in head and neck area, often in external auditory canal, sometimes on trunk or in genital region	Identical to analogous tumors of salivary glands ductal and modified myoepithelial cells forming tubular, cribriform, and solid growth patterns, infiltrative growth into surrounding tissue typically, aggregated tumor nodes with multiple mucin‐filled cysts relatively inconspicuous cytology	*Positive*: CK7, myoepithelial markers SOX10 (intensive, diffuse) and MYB (70–100 %) CD117 diffuse (> 50 %) aSMA, CK15 (36 %), vimentin (57 %)^75^	77 reported tumors: 17 x *MYB* expression 16 x *MYB* rearrangement 6 x *MYB‐NFIB* fusion 3 x *MYBL1* expression 2 x *MYBL1‐NFIB* fusion	Spread of extracutaneous adenoid cystic carcinomas of salivary glands, lacrimal glands, paranasal sinuses, larynx, bronchi, lungs, breast, cervix, Bartholin's glands, and prostate (topography!) very rarely secondary cutaneous metastasis of an extracutaneous adenoid cystic carcinoma
***Mucinous carcinoma of the skin*** ^76^, ^77^, ^78^ ***Endocrine mucin‐producing sweat gland carcinoma*** ^79,80^ (most likely to be allocated to cutaneous mucinous carcinomas)	Solitary erythematous nodes Small, rough, reddish nodes	Adults, women slightly more often	Predominantly head and neck area, especially periorbital Periorbital region, especially eyelids	Dermal, poorly defined tumor consisting of several nodes; nests, solid formations and cribriform clusters of tumor cells in abundant mucinous stroma; sometimes ductal differentiation and in situ component Circumscribed, multinodular; solid papillary cystic clusters; uniform basaloid tumor cells; small mucin lakes	*Positive*: CK7, EMA, CEA, hormone receptors; sometimes myoepithelial localization (in situ component) negative: CK20 *Positive*: Ber‐EP4, CK7, CAM 5.2, synaptophysin, chromogranin, hormone receptors, MYB, INSM1	No or no recurrent genetic alterations reported only diploid DNA with minimal CNAs, low TMB, rarely MSI, no MMR deficiency No or no recurrent genetic alterations reported	Tumors on trunk and axilla clinically potentially indicative of metastatic mucinous breast cancer similarly, metastatic mucinous colorectal carcinomas (CK20 or CDX2‐positive) in case of genital or anal localization Differentiation of basal cell carcinomas from mucinous carcinoma is difficult, indications are connection to epidermis, cleft formation to stroma, and nuclear palisading; hybrid forms are possible
***Cutaneous adenosquamous carcinoma*** ^81^, ^82^, ^83^, ^84^ (*Syn*.: squamoid eccrine ductal carcinoma)	Frequently, ulcerated node or plaque in actinically damaged skin	Especially in older men	In actinically damaged skin	Biphasic tumors with superficial squamous cell carcinoma and deep ductal carcinoma component with desmoplastic stroma infiltrative growth marked atypies, numerous mitoses perineural and lymphovascular infiltration	*Positive*: Depiction of ductal differentiation with EMA and CEA	16 reported tumors: generally high TMB UV‐associated mutations 10 x *TP53* mutations 8 x *NOTCH1* and *NOTCH2* mutations overexpressed genes: associated with sweat gland differentiation (*KRT31*, *KRT77*, *DCD*, *PIP*, *SCGB2A2*) and salivary gland differentiation (*AMY1C*, *DNASE2B*)	No ductal component in squamous cell carcinoma; no marked atypies in microcystic adnexal carcinoma; poroid cytology and absent biphasic component in porocarcinoma
***Cutaneous (apocrine) cribriform carcinoma*** ^85^, ^86^, ^87^, ^88^ (*Syn*.: cribriform tumor)	Solitary, rough nodules or nodes up to 3 cm in size with clinically benign appearance	Median patient age 47 Y; slightly more often in women	Often extremities	Deep dermal and subcutaneous, no connection to epidermis bright, eosinophilic tumor cells, minor atypia, few mitoses peripherally infiltrative growth cribriform appearance due to multiple vacuoles in individual epithelial clusters with thin, filamentous septations in ductal lumina without mucin	*Positive*: Her2 (variable), ER (variable) negative: Gata3, PR; no myoepithelial component^85^, ^86^, ^87^	No or no recurrent genetic alterations reported	May be misinterpreted histomorphologically as metastases of breast cancer or adenocarcinoma; additional DD: cutaneous secretory carcinoma
***Secretory carcinoma of the skin*** ^89^, ^90^, ^91^, ^92^	Dermal/ subcutaneous node	3rd–8th decade of life^92^ slightly more often in women	Often axillary, also head and neck area, extremities, trunk	Well‐defined, dermal and subcutaneous, no connection to epidermis inconspicuous oval, round to cuboid cells microcystic and tubular structures with abundant eosinophilic, PAS‐positive secretion, in addition solid areas focal pseudopapillary structures, rarely also mucin‐containing areas	*Positive*: S100, mammaglobin, STAT5, GATA3, NTRK	15 reported tumors: 7 x *ETV6* rearrangement 7 x *ETV6::NTRK3* fusion	Identical tumor described in mammary gland and salivary gland
***Microsecretory carcinoma of the skin*** ^91^	Small nodular tumors	Older patients		Microcystic tubules with basophilic luminal secretion	*Positive*: S100, SOX10, p63; foci with myoepithelial differentiation negative: mammaglobin	*MEF2C::SS18* fusion	Identical tumor described in salivary gland
***Signet ring cell carcinoma of the skin*** ^93^, ^94^	Diffuse eyelid swelling, sometimes with erythema (mimicking inflammatory diseases – blepharitis, chalazion); axillary circumscribed plaque or node	Median patient age 66 Y; significantly more often in men	2/3 of tumors on eyelids, 1/3 axillary	Thread‐ or lawn‐like arrangement of atypical cells with excentric nuclei and either vacuolar (signet ring variant) or eosinophilic, granular cytoplasm (histiocytic variant)	*Positive*: Ber‐EP4, androgen receptor; podoplanin, and p63 in primary signet ring carcinoma of the eyelid (but not in eyelid metastases of other tumors) rather negative: ER, PR	Mutations of *DH1*, *PIK3CA*, *NTRK3*, *CDKN1B*, and *TP53* have been described	Important differentiation from metastases of invasive lobular breast cancer (metastasis in eyelids described several times) or gastrointestinal adenocarcinoma with signet ring cell morphology – thorough examination necessary
***Eccrine ductal carcinoma NOS (represents a diagnosis of exclusion)*** ^83^	Tumor nodes and plaques	Adults		Infiltrating dermal tumors pleomorphic, polygonal, epithelioid tumor cells in, frequently, dermoplastic stroma, ductal differentiation perineural and lymphovascular infiltration, tumor necroses	*Positive*: depiction of ductal differentiation with EMA and CEA	No or no recurrent genetic alterations reported	Resembles metastatic colonization especially of breast cancer; represents a diagnosis of exclusion
***Cutaneous NUT carcinoma*** ^95^, ^96^, ^97^	Dermal/ subcutaneous node	Mostly 7th–9th decade of life^95^; women significantly more often affected	Individual cases in capillitium, face, extremities, and acral areas	Sometimes low level of differentiation with large vesicular nuclei, prominent nucleoli, mitoses, and necroses^96^ *BRD3*‐rearranged tumors show connection to epidermis, micronodular composition, and ductal structures or intracytoplasmic lumina *BRD4*‐rearranged tumors are composed of large nodes	*Positive*: NUT, p63, EMA, TRPS1, c‐MYB, CD56, CK, and INSM1 variably expressed, diffuse SOX10 in 75 %	*8 reported tumors*: *4 x BRD3::NUTM1 fusion* *3 x BRD4::NUTM1 fusion* *1 x BRD3::NUTM2B fusion*	Tumors identical to NUT carcinoma described in sinonasal, nasopharyngeal, and thoracic areas; in these locations, worse prognosis, younger patients

*Abbr*.: aSMA, alpha smooth muscle actin; Ber‐EP4, epithelial cell adhesion molecule; CAM 5.2, mouse monoclonal antibody against cytokeratin; CD117, tyrosine kinase receptor KIT; CEA, carcinoembryonic antigen; CK, cytokeratin; CNA, *copy number alterations*; CNV, *copy number variations*; DD, differential diagnosis; EMA, epithelial membrane antigen; ER, estrogen receptor; GATA3, *GATA binding protein 3*; GCDFP‐15, *gross cystic disease fluid protein 15*; GFAP, *glial fibrillary acidic protein*; HMGA2, *high mobility group AT‐hook 2*; HR, *hazard ratio*; Y, years; MAML2, *mastermind‐like transcriptional coactivator 2*; MMR, mismatch repair; MYB, *myeloblastosis*; MSI, microsatellite instability; NTRK, *neurotrophic tyrosine kinase receptor*; NUT, *nuclear protein in testis*; PLAG1, *pleomorphic adenoma gene 1*; PR, progesterone receptor; Syn., synonym; STAT5, *signal transducer and activator of transcription 5*; TMB, *tumor mutational burden*; TRPS1, *trichorhinophalangeal syndrome 1*

#### Porocarcinoma

Porocarcinomas are the most common sweat gland carcinomas. Incidences between 0.02 and 0.06 cases per 100,000 population/year are mostly reported in the literature. According to studies from England, the incidence is higher with 1.9 cases per 100,000 population/year.^2^, ^17^, ^18^, ^19^ Development in the upper portions of excretory ducts of eccrine sweat glands is suspected. In 20–30 % of porocarcinomas, an existing poroma is detected. Porocarcinomas belong to the aggressive tumors from the group of sweat gland carcinomas. A local recurrence rate of approximately 20 % has been reported, although this is lower after microscopically controlled surgery.^20^ A meta‐analysis of 453 patients from 2017 found a metastasis rate of 31 %, most often with metastasis in lymph nodes and lungs.^21^ In a publication from 1992, a mortality rate after lymph node metastasis of 67 % was reported.^22^ However, more recent data suggest lower numbers.^23^

Porocarcinomas occur predominantly in older patients and present with similar clinical features as squamous cell carcinomas. They are mostly localized on the lower extremities and in the head and neck area.^24^ In courses with metastasis formation, a pronounced lymphedema with multiple epidermotropic metastases in the form of lymphatic seeding of tumor cells may develop between primary tumor and lymph node metastasis.^25^, ^26^, ^27^, ^28^, ^29^

Histologically, porocarcinomas present with infiltrative growth of epithelial clusters of atypic poroid cells with focal ductal differentiation and numerous mitoses. While extensive tumor necroses may be present, these are not sufficient as sole criterion for malignity, given that necrosis *en masse* may also occur in poromas. The intraepithelial (in situ) form of porocarcinoma is referred to as malignant hidroacanthoma simplex and must be differentiated histologically from benign hidroacanthoma simplex as well as pagetoid Bowen's disease and clonal seborrheic keratosis. Portions with clear cell and squamoid differentiation are quite common. Colonization with melanocytes was described in approximately 20 % of cases, which may clinically present with pigmentation and thus raise suspicion of a melanocytic tumor.^24^

Despite the low penetration depth, many porocarcinomas show intralymphatic spread in the tumor periphery. Factors indicating a poor prognosis include high mitosis rate, tumor penetration depth of more than 7 (–10) mm, and evidence of intralymphatic tumor spread.^24^, ^30^, ^31^, ^32^ An infiltrative dermal pattern has been described as an additional criterion for metastasis fomration (*scattered pattern of the dermal invasive component* in contrast to the displacing *pushing* pattern).

#### Hidradenocarcinoma

Besides porocarcinoma, hidradenocarcinomas are also common sweat gland carcinomas affecting middle‐aged patients without clear gender prevalence. The capillitium, neck, and face are the most common localizations, followed by the extremities.^33^ The tentative clinical diagnosis is usually a cyst. The tumors may arise from an existing hidradenoma or *de novo*. In the literature, metastasis formation was mostly described within the first two years in 36 %–60 % of the patients.^34^, ^35^ In cases with metastasis, lymphatic spread is paramount. Risk factors include tumor size, in particular, as well as poor differentiation and perineural invasion. In a study from 2015, no metastasis formation was observed in ten patients with hidradenocarcinoma during a median postoperative follow‐up period of 7 years.^33^ According to another study by Gao et al. including almost 300 hidradenocarcinomas, the prognosis is significantly better than previously assumed.^36^ Distant metastases were only present in 2.4 % of cases. The tumor‐specific 10‐year survival was 90.5 %. The only factor affecting tumor‐specific survival was the tumor size, in contrast to localization, gender, age, ethnicity, grade of histological differentiation, or type of surgical treatment.^36^

In histology, a nodular, dermal to subcutaneous tumor with sections revealing a pre‐existing hidradenoma can be found. Cell atypia, infiltrative tumor growth, and tumor necroses are observed. A high number of mitoses (Ki‐67 > 11 %), in particular, is considered evidence of hidradenocarcinoma.^36^ Clear cell and mucinous portions as well as squamous metaplasia may be present. Given that the carcinomatous development may occur in foci of existing hidradenomas, complete processing of the tumors is recommended in hidradenomas. Nevertheless, clearly benign hidradenomas with exclusive metastasis to lymph nodes have been described in very rare cases. Similar to some Spitz tumors or metastatic leiomyomas of the uterus, these are probably part of a spectrum of tumors presenting with lymph node metastases but not spreading further.^37^ In five patients with clear cell hidradenoma, there was no case of metastasis formation after surgical excision within a follow‐up period of 2 to 11 years. Clear cell hidradenomas with intralymphatic tumor cells should be referred to as “atypical clear cell hidradenomas”, and affected patients should be monitored more closely. Moreover, atypical hidradenomas should be excised completely, given the difficult histopathological differentiation from early hidradenocarcinoma. Furthermore, differentiation from hidradenoma is also impeded in well‐differentiated hidradenocarcinomas.^38^ In particular, superficial or partial excision may result in misdiagnosis.^38^ In tumors with the histomorphology of hidradenocarcinoma localized in the axillary region, differential diagnosis must rule out metastasis or an extension of a primary breast cancer.^39^, ^40^

#### Extramammary Paget's carcinoma

Extramammary Paget's carcinoma (also, extramammary Paget's disease) is a rare intraepithelial sweat gland carcinoma with apocrine differentiation arising primarily in inguinal, perianal, perigenital, and axillary regions. Initally, genital localizations, in particular, are clinically often misdiagnosed as inflammatory dermatosis.

In differential diagnosis, histopathological findings of Paget's carcinoma in the anogenital region require awareness of potential spreading of colorectal and urogenital carcinomas *per continuitatem* and necessitate corresponding examinations. According to a recent guideline of US‐American colleagues based on retrospective data, screening for other adenocarcinomas should be performed according to the anatomical subtype.^41^ Thus, perianal Paget's carcinoma has been associated with a high rate of adenocarcinomas (25 %, mainly colorectal), but penoscrotal and vulvar Paget's carcinomas were associated with only 6 % (mainly urogenital). A screening algorithm has been proposed including coloscopy, urine cytology, and computed tomography of chest, abdomen, and pelvis in perianal Paget's carcinoma, and urine cytology, heme‐occult test, and test for prostate specific antigen (especially in patients under 70 years of age) in penoscrotal Paget's carcinoma. In vulvar Paget's carcinoma, urine cytology and mammography are recommended.

Histology is characterized by intraepithelial localization of individual larger, bright cells (Paget cells), sometimes forming small groups (nests), with PAS‐positive and sometimes mucin‐containing cytoplasm that may spread invasively. Ductal structures within the nests and positioning with preservation of the keratinocytic layer of basal cells are quite typical. The lateral margin in Paget's carcinoma is very poorly defined (*skipped areas*); moreover, spreading along adnexal structures may occur, complicating an assessment of the completeness of the excision and explaining the high recurrence rates.^42^ In immunohistochemistry, the cells of extramammary Paget's carcinoma are labeled by CK7 and CAM 5.2. This allows for differentiation from pagetoid Bowen's disease in most cases and should also be used for margin control, if appropriate. Given that melanomas may also exhibit pagetoid spread in the epidermis while Paget cells may sometimes mimic nest‐like structures, immunohistochemical staining with melanocytic markers is recommended for differentiation. Moreover, immunohistochemical markers, such as CK20 and CDX2, may indicate another underlying carcinoma.^43^, ^44^, ^45^, ^46^

#### Microcystic adnexal carcinoma

Microcystic adnexal carcinoma is a slowly growing, locally strongly infiltrating sweat gland carcinoma of low malignancy and potentially also a tumor with both follicular and sweat gland differentiation. The incidence is reported as 1.6 to 6.5 cases per 10,000,000 population per year. The tumors arise predominantly in the centrofacial area of older patients, women are slightly more often affected.^47^ However, manifestation already from the 3rd decade of life has been described.^48^, ^49^ Predilection sites include predominantly the head and neck area, mainly the face and here primarily the cheeks. Occasionally, microcystic adnexal carcinomas are found on the trunk and very rarely on the extremities. The plaques are indurated on palpation and may have a diameter of several centimeters; clinically, the size is usually underestimated due to the infiltrative growth. Clinically, the tumors resemble infiltrative basal cell carcinoma. The carcinomas infiltrate deeply into the subcutis, often forming perineural tumor extensions that are one cause of the high local recurrence rate in cases with inadequate excision. Microscopically controlled excision or excision with a safety margin is required. Metastasis formation is an extremely rare event that is only observed in immunosuppressed patients, and then only to the locoregional lymph nodes.^50^, ^51^

Histopathologically, microcystic adnexal carcinoma is characterized by clusters of tumor cells with dermal or subcutaneous localization without connection to the epidermis. In the upper portions of the tumor, larger, cystically dilated sweat gland excretion ducts are observed, reminiscent of small infundibular cysts (milia). In contrast, the deeper portions, partly reaching into the subcutis, are characterized by small syringoid clusters of epithelial cells without cytomorphological abnormalities or mitoses and elongated tubular structures in hyaline, sclerotic, or mucinous connective tissue. Perineural spread in the tumor periphery is typical. Patches of lymphocytic infiltrates are observed. No established immunohistochemical markers for distinguishing the histological differential diagnoses are available. Microcystic adnexal carcinoma is either Ber‐EP4‐negative or shows only focal staining of membranes. Overall, infiltration of the subcutis and perineural spread present the most important diagnostic evidence for differentiation from syringoma and desmoplastic trichoepithelioma, in particular. In some cases, the absence of CK20‐positive Merkel cells may help in differentiation from desmoplastic trichoepithelioma. An important aspect is the differentiation from syringoid hamartomatous proliferations in some familial syndromes, such as Rombo syndrome and Nikolau‐Balus syndrome. In these usually younger patients, plaques with clinical features reminiscent of microcystic adnexal carcinoma are present on both cheeks. In the face, these are mostly accompanied by telangiectasias, milia‐shaped cysts, and/or atrophoderma vermiculata; these changes may also be present on the upper arms of the patients. However, there is no evidence of the perineural infiltration typical of microcystic adnexal carcinoma. Correct diagnosis using a representative tissue biopsy is essential to avoid subsequent extensive mutilating surgical interventions.^52^, ^53^ In microcystic adnexal carcinoma, four genes of the calcium signaling pathway are upregulated on RNA and protein level: *CACNA1S*, *ATP2A1*, *RYR1*, and *MYLK3*, which may serve as diagnostic markers in the future.

#### Digital papillary adenocarcinoma

Digital papillary adenocarcinoma is a disease of adulthood with a peak in the 5th decade of life affecting men more frequently than women.^54^, ^55^ Individual cases of significantly younger patients have, however, been described. Clinically, slowly growing, solitary nodes are found on fingers and toes, sometimes on soles and palms. While digital papillary adenoma was initially distinguished from digital papillary adenocarcinoma, it was later found that a reliable prognosis is not possible based on histological features and grade of differentiation.^54^, ^56^ In a recently conducted larger study, human papillomavirus 42 (HPV42), which had previously been classified as *low‐risk* HPV type, was detected in 96 % of the analyzed digital papillary adenocarcinomas.^8^, ^57^ The HPV42‐induced transformation triggers a germ cell‐like transcription program that is conserved in all HPV‐positive carcinomas and, essentially, represents a “fingerprint” of oncogenic HPV. Based on the detection of HPV42, digital papillary adenocarcinomas were increasingly found in non‐acral localizations as well, expanding the morphological spectrum of these neoplasms.

Digital papillary adenocarcinoma is prone to local recurrence. According to literature data, metastases occur in 14 to 41 % of patients, often only after several years and predominantly affecting lymph nodes and lungs.

Histologically, a dermal to subcutaneous tumor without connection to the epidermis and cystic to solid cystic structure is observed.^56^ On first examination, the majority of tumors present inconspicuous and harmless with well‐defined nodular outlines. The predominantly basophilic tumor cells show moderate cytological atypies, and often form papillary structures as well as back‐to‐back stratification of neoplastic formations. However, the papillary structures may be completely absent. Usually, several mitoses are found. Squamous metaplasia and clear cell differentiation may occur. Ultimately, there is a broad morphological spectrum ranging from hidradenoma‐ or cystadenoma‐like appearance to highly pleomorphic infiltrative epithelial proliferations. The tumors may be completely solid or extremely cystic, thus resembling hidrocystomas that virtually never occur in acral areas.

HPV42 detection is very helpful in differential diagnosis. Immunohistochemically, the central glandular cell clusters show inhomogeneous staining of CK7. Increased proliferation (Ki‐67) is detectable. A typical feature is a compartmentation with the detection of a second population of myoepithelial cells, enabling differential diagnosis from, for example, nodular hidradenoma.

#### Adenoid cystic carcinoma

Adenoid cystic carcinoma is a rare cutaneous adnexal tumor slightly more often diagnosed in women, typically in the 6th decade of life.^58^, ^59^ It is predominantly localized in the head and neck area, often in the external auditory canal, occasionally on the trunk or in the genital area.^60^ Local recurrence may occur, lymph node metastases and distant metastases (especially lungs) are extremely rare but may appear even after many years.^61^, ^62^, ^63^ However, tumors in the external auditory canal have a higher risk of metastasis of approximately 30 % similar to the salivary gland tumors of the same name. A higher recurrence and metastasis rate was also reported for the genital area. In one study on 201 patients, the 5‐year and 10‐year survival was reported as 87.0 % and 76.0 %, respectively.^59^ In 65.8 % of the patients, a localized tumor was diagnosed (stage I and II).

Histologically, cutaneous adenoid cystic carcinoma is identical to analogous tumors of salivary glands. Ductal and modified myoepithelial cells forming tubular, cribriform, and solid growth patterns are identified. Typical features are aggregated tumor nodes with multiple mucin‐filled cysts (adenoid cystic pattern) and a relatively inconspicuous cytology. The tumors may grow in an infiltrative manner into the surrounding tissue. The recurrence rate is increased in cases with perineural spread (76 %).

In immunohistochemistry, expression of myoepithelial markers is usually found in addition to expression of CK7. A *MYBL1* fusion has been identified by molecular genetic analysis.^64^, ^65^ A 1p36 deletion, considered a marker for a poor prognosis in adenoid cystic carcinomas of salivary glands, has not yet been identified in cutaneous adenoid cystic carcinomas.^66^

Depending on the localization, cutaneous spread of an extracutaneous glandular tumor needs to be excluded in differential diagnosis. Apart from salivary glands, extracutaneous adenoid cystic carcinomas arise also in lacrimal glands, paranasal sinuses, larynx, bronchi, lungs, breast, cervix, Bartholin's glands, and prostate. However, cutaneous metastasis of an extracutaneous adenoid cystic carcinoma presents an absolute rarity.^67^

### Genetic alterations

*Chapter authors*: Silke Redler, Lara Racz, Friedegund Meier, Jürgen C. Becker, Almut Böer‐Auer

Sweat gland carcinomas also represent a heterogeneous group of rare tumors with respect to their genetics. Although a monogenic inheritance with germ‐line mutations cannot completely be ruled out in individual families (for example, in Brooke‐Spiegler syndrome), it must be assumed that sweat gland carcinomas are predominantly sporadic tumors not arising in the context of an inherited tumor risk syndrome. The development of sporadic tumors is associated with a multi‐stage process with cumulative acquisition of somatic mutations in different genes as well as other complex genomic alterations.

Table 1 summarizes the most important genetic changes in various entities of sweat gland carcinomas and the somatic therapeutic targets identified to date. Only recurrent genetic findings are listed.

While extensive molecular genetic insights are available for other malignant skin tumors, the molecular genetic knowledge on sweat gland carcinomas is so far limited. Only for individual entities does a more comprehensive picture exist, giving insight into the pathogenesis and classification of these tumor entities and thus providing the option of targeted therapeutic approaches and individualized precision medicine. For example, specific molecular alterations – especially in eccrine porocarcinoma – have already been used for promising therapeutic approaches.

Precision therapy is increasingly gaining importance in tumor therapy, especially for rare tumor entities, such as sweat gland carcinomas, for which no evidence‐based, guideline‐conforming therapeutic standards are available to date.

The molecular examination methods utilized in sweat gland carcinomas are summarized in Table 1. They are inhomogeneous and range from the determination of individual genetic variants and the specific analysis of single, clearly defined regions to extensive genetic analyses with so‐called gene panels or whole exome analyses (whole exome sequencing, WES) and various techniques for gene expression profiling.

While the number of tumors examined in the respective studies differs, it is generally low compared to more common tumor entities. In some studies, only the tumor DNA (tumor‐only analysis) was examined, while in other studies tumors and constitutional variants (usually in the form of adjacent tumor‐free skin) were analyzed in parallel. The analyzed collectives are too small for a final assessment of intergenetic and intragenetic heterogeneity of these tumor entities.

It is a key objective to utilize the methodological developments in the form of perspective whole genome profiling of tumors also for sweat gland carcinomas. These analyses should be performed in a quality‐assured setting and include a process comprising pre‐analysis of sample material, sequencing, identification, annotation, and classification as well as clinical interpretation of variants. A relevant aspect is the integration into a clinical context, including indication, consulting services and, where appropriate, consultations with the interdisciplinary organ board or the molecular tumor board. This includes a thorough understanding of the clinical trial landscape and current regulatory status. Integration of human genetics is desired. With the increasing use of comprehensive analysis of both tumor DNA and adjacent normal tissue, the likelihood of detecting variants that may represent inherited (constitutional) variants is increasing. This may necessitate counseling of patients and their relatives in compliance with the German Genetic Diagnostics Act by a specialist in human genetics.

### Dermoscopy and other methods of in vivo imaging

*Chapter author*: Michael Erdmann

#### Dermoscopy

There are no established diagnostic, dermoscopic criteria for the various sweat gland carcinomas that would allow for a specific diagnosis and/or differentiation from their benign counterparts or clinical differential diagnoses. Based on case reports summarized in a review, sweat gland carcinomas have dermoscopic patterns that may mimic basal cell carcinoma, cutaneous squamous cell carcinoma, melanocytic tumors, or other benign skin tumors.^98^

#### Optical coherence tomography (OCT), line‐field confocal optical coherence tomography (LC‐OCT), and reflectance confocal microscopy (RCM)

Case reports or small case series on sweat gland carcinoma are available for these high‐resolution imaging techniques.

In reflectance confocal microscopy (RCM), a recurrence of porocarcinoma presented with nests of poroid cells in addition to correlates of ductal differentiation with surrounding tortuous, elongated vessels in a dark stromal background.^99^

In a case series, extramammary Paget's carcinoma presented with characteristic changes with histopathological correlate, such as eliminated honeycomb pattern, intraepithelial Paget cells, basal ovoid nests, inflammatory cells, and tortuous vessels in RCM.^100^ Moreover, individual Paget cells with dark cytoplasm and bright nucleus could be distinguished from adjacent keratinocytes by line‐field confocal optical coherence tomography (LC‐OCT).^101^ In case reports of microcystic adnexal carcinomas, the dermis presented with round, in part onion‐like, cystic areas in addition to linearly arranged ductal structures in OCT, whereas tumor islands with clefting and adjacent fibrosis were observed in RCM.^102^, ^103^

#### Sonography, computed tomography (CT) und magnetic resonance imaging (MRI)

While sonography, computed tomography (CT), and magnetic resonance imaging (MRI) will usually not provide any additional information relevant for differential diagnosis of primary sweat gland carcinomas, they may facilitate estimation of the infiltration depth prior to surgery and may be helpful in distinguishing between histomorphologically identical tumors, such as metastases of breast cancer or adenocarcinomas. They are the imaging techniques of choice for primary staging and follow‐up, particularly for assessing the regional lymph node status and ruling out distant metastases.

## PROGNOSIS AND STAGING

*Chapter author*: Carsten Weishaupt

On 1 January 2026, the 9th edition of the UICC TNM system came into effect. This edition includes adnexal carcinomas of the skin in the TNM classification for ‘skin carcinomas’ (see Table 2). The classification for tumours in the head and neck region differs only in the N classification (Table 3).

**TABLE 2:** TNM‐classification of carcinomas of the skin (apart from eyelid, head‐and‐neck, vulva, penis and perianal skin); pT‐ and pN‐categories correspond to the cT‐ and cN‐categories

**T – primary Tumour**	
cTX	Primary tumour cannot be identified
cT0	No evidence of primary tumour
cTis	Carcinoma in situ
cT1	Tumour 2 cm or less in greatest dimension
cT2	Tumour > 2 cm and ≤ 4 cm in greatest dimension
cT3	Tumour > 4 cm in greatest dimension or minor bone erosion or perineural invasion or deep invasion*
cT4a	Tumour with gross cortical bone / marrow invasion
cT4b	Tumour with axial skeleton invasion including foraminal involvement and/or vertebral foramen involvement to the epidural space
* “Deep invasion” is defined as invasion beyond the subcutaneous fat or > 6 mm (as measured from the granular layer of adjacent normal epidermis to the base of the tumour). Perineural invasion is defined as tumour cells within the nerve sheath of a nerve lying deeper than the dermis or measuring 0.1 mm or larger in caliber or involvement of five or more nerves per section. In the case of multiple simultaneous tumours, the tumour with the highest T category is classified and the number of separate tumours is indicated in parentheses, e.g., T2(5).	
**N – Regional Lymph Nodes**	
cNX	Regional lymph nodes cannot be assessed
cN0	No regional lymph node metastasis
cN1	Metastasis in single regional lymph node 3 cm or less in greatest dimension
cN2	Metastasis in a single ipsilateral lymph node, more than 3 cm but not more than 6 cm in greatest dimension, or in multiple ipsilateral lymph nodes not more than 6 cm in greatest dimension
**M – Distant Metastasis**	
M0	No distant metastasis
M1	distant metastasic disease*
* Contralateral lymph nodes in non‐melanoma non‐head and neck cancer are distant metastases.	

**TABLE 3:** clinical N‐classification of head‐and‐neck carcinomas of the skin (apart from eyelid)

**N‐Classification**	
cNX	Regional lymph nodes cannot be assessed
cN0	No regional lymph node metastasis
cN1	Metastasis in a single lymph node 3 cm or less in greatest dimension
cN2a	Metastasis in a single ipsilateral lymph node, more than 3 cm but not more than 6 cm in greatest dimension without extranodal extension
cN2b	Metastasis in multiple ipsilateral lymph nodes, none more than 6 cm in greatest dimension, without extranodal extension
cN2c	Metastasis in bilateral or contralateral lymph nodes, none more than 6 cm in greatest dimension, without extension extension
cN3a	Metastasis in lymph nodes, more than 6 cm in greatest dimension, without extranodal extension
cN3b	Metastasis in a single or in multiple lymph nodes with clinical with extranodal extension*
*Clinical extranodal extension is defined as the presence of skin involvement or soft tissue invasion with deep fixation to underlying muscle or adjacent anatomical structures or clinical signs of nerve involvement (e.g., cutaneous/subcutaneous satellite or in‐transit metastases).	
**Pathological N‐classification of carcinomas of the skin in head‐and‐neck localization** (apart from eyelid)	
pNX	Regional lymph nodes cannot be assessed
pN0	No regional lymph node metastasis
pN1	Metastasis in a single ipsilateral lymph node, 3 cm or less in greatest dimension without extranodal extension
pN2a	Metastasis in a single ipsilateral lymph node, less than 3 cm in greatest dimension with extranodal extension or, more than 3 cm but not more than 6 cm in greatest dimension without extranodal extension
pN2b	Metastasis in multiple ipsilateral lymph nodes, not more than 6 cm in greatest dimension without extranodal extension
pN2c	Metastasis in bilateral or contralateral lymph nodes, none more than 6 cm in greatest dimension, without extension extension
pN3a	Metastasis in a lymph node more than 6 cm in greatest dimension without extranodal extension
pN3b	Metastasis in a lymph node more than 3 cm in greatest dimension with extranodal extension or multiple ipsilateral, or any contralateral or bilateral node(s) with extranodal extension
Pathological extranodal spread should only be diagnosed when a tumour that extends entirely within a lymph node penetrates through the entire thickness of the lymph node capsule into the surrounding connective tissue, with or without a stromal reaction. A soft tissue mass at a site where a lymph node would be expected to be located should be considered as at least one lymph node with extranodal spread.	

**TABLE 4:** Stages of skin carcinomas inclusively head‐and‐neck region (apart from eyelid)

**Stadium 0**	Tis	N0	M0
**Stadium I**	T1	N0	M0
**Stadium II**	T2	N0	M0
**Stadium III**	T3 T1, T2, T3	N0 N1	M0 M0
**Stadium IVA**	T1, T2, T3 T4	N2, N3 Any N	M0 M0
**Stadium IVB**	Any T	Any N	M1

Tumours of the eyelids, vulva, penis and perianal skin are classified separately according to their specific location. For these locations, please refer to the comprehensive classifications in the 9th edition of the UICC TNM classification.

Involvement of the adjacent locoregional skin or soft tissues (e.g., cutaneous/subcutaneous satellite or in‐transit metastases) is not explicitly described. In the N category for skin carcinomas in the head and neck region, such involvement is classified as “extranodal” extension and assigned an N3b stage (Table 4).

Primarily, sweat gland carcinomas are locally progressive.

In addition to environmental (access to specialized medical care) and socioeconomic factors, the 9th edition of the UICC TNM classification lists clinical and histopathological prognostic factors; however, these have not yet been systematically evaluated for sweat gland carcinomas.

Clinical prognostic factors for progression or recurrence include:

- TNM stage (e.g., regional lymph node metastases or distant metastases at the time of initial diagnosis)
- Location, tumours extending to the orbit/sinuses
- Immunosuppression
- Age
- Recurrent disease
- Growth rate

Histological prognostic factors include:

• Histopathological type
• Tumour thickness
• Differentiation
• Perineural invasion
• Lymphovascular invasion

In general, positive surgical margins, large and thick tumours, perineural invasion, high mitotic rates, and/or lymphatic vessel invasion are risk factors for local recurrence and/or metastasis in most types of cancer. Clinically, immunosuppression or concomitant hematoproliferative disorders play a particularly significant role in more aggressive disease courses.

Given the rare diagnosis and the inadequate data available, no valid staging exists for most subtypes of sweat gland carcinomas. Overall, the classification into local, lymph node and distant metastatic stages analogous to other cutaneous carcinomas appears reasonable. In this context, the local stage refers to tumors confined to the tissue of origin, the nodal stage to histologically confirmed regional lymph node metastases. Sweat gland carcinomas are primarily locally progressive. In the literature, porocarcinoma and microcystic adnexal carcinoma are often classified according to the AJCC classification for cutaneous squamous cell carcinoma. General risk factors for progression or recurrence have not yet been evaluated. In principle, positive excision margins, large and thicker tumors, perineural invasion, high mitosis rate, and/or lymphovascular invasion are risk factors for local recurrence and/or metastasis in most entities. From a clinical perspective, immunosuppression or hematoproliferative comorbidities, in particular, play a role in more aggressive courses.

Table 5 presents the prognosis and prognostic factors of subtypes of sweat gland carcinomas described in the literature; the stated numbers are based on multiple case series and cohorts (Table 5).

**TABLE 5:** Prognosis and prognostic factors.

Subtype	Prognosis	Prognostic risk factors
Porocarcinoma^20^, ^104^	5YSR overall 74.8 % 5YSR st I+II 96 % 5Y local recurrence rate 11.5 % 5Y local recurrence rate st I+II: 20 % (excision margins 2–3 cm) 31 % metastasis (58.5 % LN, 12.8 % lungs; liver, brain possible) 8.1 % metastasis already at diagnosis 67 % mortality in case of LN metastasis, then 1YSR 84 %, 2YSR 32 %	− > 14 mitoses/HPF − tumor penetration depth > 7 mm − clear cell differentiation − infiltrative and pagetoid porocarcinoma − lymphovascular invasion − advanced age
Hidradenocarcinoma^105^	1YSR 98.2 %; 3YSR 97.2 %, 5YSR 93.8 %, 10YSR 90.5 % (all tumor‐specific) 2.4 % metastasis (recent analyses), predominantly lymphatic, mainly within two years (of these, 2.1 % already at diagnosis; after LN, also lungs, liver, brain, bones)	Overall survival and tumor‐specific survival mainly dependent on tumor size
Malignant mixed tumor of the skin^106^	Local recurrence rate: 19.15 % (due to cutaneous extensions) 60 % metastasis (LN, lungs, pleura, brain, bones, liver, bone marrow, kidneys, thyroid) 19.15 % metastasis at diagnosis 25 % mortality worse prognosis for myoepithelial carcinoma	
Spiradenocarcinoma^107^, ^108^, ^109^	5YSR 77 % time to local recurrence/metastasis: 20.1 mo (0–92 mo) 24‐month RFS: 37.2 % (45/121) 24‐month mortality rate: 10.2 % (13/121) median survival: 16 months metastasis 37.4 % (lungs (34.7 %), bones (26.5 %), LN (53.1 %), liver (14.3 %), brain (10.2 %), skin (10.2 %), kidneys, bone marrow, large arteries, and pelvis 4.1 % each, mediastinum and intestine 2 % each local recurrence: 20.8 % mortality: 19.1 %	Brooke‐Spiegler syndrome
Cylindrocarcinoma^110^, ^111^	Metastasis in 18/< 50 cases described in literature mortality rate: 35.5 % (11/31)	Brooke‐Spiegler syndrome
Syringocystadenocarcinoma papilliferum^112^	8 % recurrence (4/50) 20 % metastasis: LN, lungs, spinal column, subcutaneous tissue	Nevus sebaceus
Extramammary Paget's carcinoma^113^, ^114^	Local recurrence 33–40 % despite extensive surgical interventions prognosis predominantly dependent on localization and whether primary or secondary Paget's carcinoma (mortality of secondary extramammary Paget's carcinoma: 50 %) 5YSR tumor‐specific: 87 % (in case of local recurrence 92 %, in case of regional metastasis 77 %, in case of distant metastasis 16 %) 1 LN metastasis → 5YSR tumor‐specific: 80 % ≥ 2 LN metastases → 5YSR tumor‐specific: 40 % metastasis: up to 17 %	− Tumor penetration depth > 1 mm − age > 65 years − localization of tumor (perianal worse than trunk or scrotum, vaginal worse than vulval or labial, mucosal involvement → more LN metastases, local recurrences) − R1 resection → local recurrence risk − lymphovascular invasion → risk of locoregional and distant metastasis − HER2+ → associated with high tumor penetration depth and LN metastasis
Microcystic adnexal carcinoma^115^	Recurrence rate 4.3 % after MCS (usually in the first 2–3 years) disease‐specific 5Y mortality after MCS 2 % overall survival 10.8 years stage‐dependent overall survival: 5/10YSR st I 86 %/77 %, st II 77 %/48 %, st III 65 %/44 % in 68.5–74 % only the skin is affected, in 9–18.5 % tissue adjacent to the skin, such as subcutaneous adipose tissue, muscles, and bones, are affected; metastasis is found in up to 1 % of cases	− Risk of disease increases with age (mean age at onset 63 years SD ± 15.8) − recurrence risk increases by 11 % per 1 cm^2^ of tumor diameter; recurrence risk for tumor diameter of 5.0 cm^2^ or more increased 13‐fold − perineural invasion − UV exposure − preceding radiotherapy − aggressive courses described in patients with immunosuppression
Digital papillary adenocarcinoma^116^, ^117^	Local recurrence rate: 21–50 % at initial diagnosis: 3–5 % LN metastasis metastasis: 14–41 % (up to 20 years after initial diagnosis), 83 % in regional LN, 71 % lungs	
Adenoid cystic carcinoma^118^, ^119^	5YSR 96.1 % local recurrence rate: 44 % (in case of incomplete excision and perineural growth; sometimes many years after initial diagnosis) 25 % regional metastases 5 % distant metastases (up to 18 years after initial diagnosis), predominantly LN, lungs, also chest wall, pericardium, bones, nose, and liver higher risk in case of tumors in external auditory canal and genital area as well as perineural growth	Slightly better survival in case of men, patients < 50 years, and head and neck tumors
Mucinous carcinoma of the skin^76^, ^77^ Endocrine mucin‐producing sweat gland carcinoma^79^	7 % local recurrence rate after Moh's surgery/frozen sections PFS: 36.5 months 10.5 % metastasis in regional LN; 5.8 % in other organs, e. g. lungs local recurrence rate: 8.4–14.3 % metastasis 7.1 % (parotid gland, LN, salivary glands, lungs, bones, pancreas)	Localization on trunk or eyelid associated with worse course based on analysis of case reports
Cutaneous adenosquamous carcinoma^82^, ^83^, ^84^, ^120^	5YSR 30 % 25 % local recurrence rate 13 % metastasis (LN, lungs, bones)	
Cutaneous (apocrine) cribriform carcinoma^88^, ^121^, ^122^	No recurrence/metastasis risk two cases of persistent disease after incomplete removal reported	
Secretory carcinoma of the skin^90^, ^123^, ^124^ Microsecretory carcinoma of the skin^91^	Overall indolent in a collection of 15 cases with a follow‐up of up to 144 months, diagnosis of 1 pulmonary metastasis So far, 10 primary cutaneous cases described, none of these with recurrence or metastasis	
Signet ring cell carcinoma^93^ ^.^ ^94^	Divergent data in the literature: eyelid tumors aggressive, locally infiltrative and metastatic behavior (42 % LN and distant metastases) axillary tumors less often mainly LN metastasis other case series: 19 % LN metastases in eyelid tumors vs. 72 % in axillary tumors	
NUT carcinoma^97^	Until 2025, 17 cases with primary cutaneous NUT described: 5 patients developed LN metastases; no distant metastases described	Extracutaneous NUT have a very poor prognosis with a mean survival of 6.7 months

*Abbr*.: HPF, *high power field*; YSR, year survival rate; LN, lymph node; MCS, microscopically controlled surgery; RFS, recurrence‐free survival

## THERAPY

### Surgical therapy

*Chapter authors*: Cornelia Erfurt‐Berge, Jochen Utikal

Surgical therapy of sweat gland carcinomas is usually based on complete surgical excision of the tumor. For sweat gland carcinomas in general, microscopically controlled surgery (MCS) is recommended as therapeutic standard, especially for periocular tumors and other surgically difficult localizations.^125^ Wide local excision (WLE) presents an alternative to removing tumors by MCS – especially in non‐facial localizations, although the recurrence rates are higher.^126^ For WLE, a safety margin of 1 cm is appropriate. Periocular tumors require particular attention due to their special functional and anatomical features.^80^, ^127^

Given the unclear data, the surgical procedure should, in general, be established for each individual case depending on the specific entity and localization (Table 6).

**TABLE 6:** Recommended surgical margins for primary excision and local recurrence.

	Safety margin
Sweat gland carcinomas in general	Microscopically controlled surgery recommended; alternatively, wide local excision with safety margin of at least 1 cm^*^
Extramammary Paget's carcinoma	Microscopically controlled surgery recommended; alternatively, preoperative mapping biopsies in combination with subsequent wide local excision
Microcystic adnexal carcinoma	Microscopically controlled surgery recommended; alternatively, wide local excision with safety margin of at least 2 cm^*^
Digital papillary adenocarcinoma	Microscopically controlled surgery recommended; normal excision or amputation of finger^*^

* With significantly higher recurrence rates

In *extramammary Paget's carcinoma*, the recurrence rate after WLE is very high with 37.0 %.^128^ Large safety margins of 4 cm for penoscrotal and vulval areas as well as 3.5 cm for perianal and axillary localizations, result in complete excision of the tumor in 95 % of cases.^128^ Given that these large safety margins are not always possible for anatomical reasons, methods for margin control, such as MCS, are recommended.^129^ MCS, including Moh's micrographic surgery, achieves a lower recurrence rate of 18.7 %.^128^, ^130^ However, the circumferential assessment of peripheral and deep margins is labor‐intensive and requires many tissue sections. Therefore, performing pre‐operative mapping biopsies (PMBs) in combination with subsequent WLE is recommended. This approach resulted in a 5‐year recurrence rate of 11.7 %, whereas WLE without PMBs resulted in a recurrence rate of 45.5 %.^129^

*Microcystic adnexal carcinomas* show an aggressive local growth behavior and are often prone to recurrence. A retrospective analysis by Thomas et al. (2007) on 25 patients treated by MCS showed a recurrence rate of 12 %.^131^ In an analysis of 44 cases (including 31.8 % recurrences), Leibovitch et al. (2005) identified perineural invasion in 17.5 %.^132^ One of 20 patients presented with recurrence during a 5‐year follow‐up. There is currently no clear evidence concerning the safety margin. Each case should be treated by MCS or, alternatively, WLE with a safety margin of 2 cm, if possible.^133^

Given that *digital papillary adenocarcinoma* is prone to local recurrence, careful surgical treatment is the most important therapeutic intervention. According to a systematic review, no recurrence was observed after MCS, whereas recurrences occurred in 34.1 % after normal excision and in 20 % after finger amputation.^134^

The available evidence on the benefit of sentinel lymph node biopsy (SLNB) in sweat gland carcinomas is currently limited. Overall, positive SLNs are only rarely found in early tumor stages.^135^ Of 41 patients with sweat gland carcinoma diagnosed over a period of 19 years, 25 (61 %) had undergone SLNB. Only one of these patients showed SLN involvement. In a small retrospective histopathological study, six cases with high‐risk (invasion into subcutis, intralymphatic tumor cells detected in primary tumor) sweat gland carcinomas (hidradenocarcinoma, eccrine ductal carcinoma, porocarcinoma) that underwent SLNB, were reanalyzed. In four of the six cases, lymph node metastases of the sweat gland carcinoma were already detected by hematoxylin and eosin staining.^136^ Similar to other tumor types, immunohistochemistry seems to facilitate detection of small microscopic metastases in SLNs. Currently, there are insufficient data for SLNB as staging tool for patients with sweat gland carcinomas. Lymph node dissection may be considered in patients with positive SLNs or if lymph node metastasis is clinically suspected. The decision whether lymph node dissection is performed should be made for each individual case and discussed in the framework of an interdisciplinary tumor conference.

### Radiotherapy

*Chapter authors*: Clemens Seidel, Alexander Frisman

For each individual case, a therapeutic decision should be made in an interdisciplinary manner in the framework of a tumor board discussion taking the present clinical features, patient‐specific factors, (post)‐operative risk factors, and potential contraindications into account.

In view of the small number of prospective studies, a recommendation for radiotherapy based on high evidence grade is often difficult. The literature on individual entities is often limited to small case series or retrospective monocentric observations.^137^, ^138^, ^139^, ^140^, ^141^, ^142^

With respect to specific clinical and histological entities, the following radiotherapeutic concepts are used analogous to other sites of manifestation.

*Extramammary Paget's carcinoma*: Postoperative radiotherapy may be indicated in the following cases: Non‐invasive tumor and high morbidity due to radical tumor excision or presence of risk factors, such as large, recurrent, or multifocal tumor, residual tumor (R1–R2), invasive tumor, lymph node metastasis, positive excision margin, or elevated Ki‐67 %. For macroscopic tumors, a total dose (TD) of more than 60 Gy (EQD2) is recommended. In the adjuvant situation, 60 Gy are used in the tumor bed area and 45–50 Gy TD (EQD2) in the area of lymphatic drainage. In the current US‐American guideline for extramammary Paget's carcinoma, target volumes and dosage plans are described in detail.^143^

*Hidradenocarcinoma*: Given that the recurrence rate after resection alone is up to 50 % in poorly differentiated (high‐grade) tumors, postoperative radiation should be considered in contrast to low‐grade tumors where local recurrence after R0 resection is less common.^144^, ^145^ Due to the resistance of hidradenocarcinomas to radiation, radiotherapy with higher dosage is recommended with a TD varying between 45 and 70 Gy depending on the localization.^146^

*Microcystic adnexal carcinoma*: A Canadian guideline on this entity was published in 2019.^133^ Primary radiotherapy is recommended for inoperable tumors. Adjuvant radiation is indicated in N1, R1, or Rx findings. The aim should be a TD of 60 Gy to 66 Gy (EQD2).

*Porocarcinoma*: If risk factors exist, adjuvant radiation should be considered. Given the systemic tendency for metastasis formation, the areas of lymphatic drainage were usually included in case series, even without evidence of lymph node involvement. While very heterogeneous data from 24–70 Gy are available concerning the TD, a dose of more than 50 Gy (EQD2) was used in most cases. Accordingly, radiotherapy with a TD of 50–70 Gy (EQD2) is recommended.^146^

*Adenoid cystic carcinoma*: Analogous to adenoid cystic carcinoma of salivary glands in the head and neck area, a TD of up to 70 Gy (EQD2) is recommended for macroscopic tumors. For the adjuvant situation, doses of 66 Gy in R1 resection and 50–60 Gy in large (pT3/T4) R0‐resected tumors or in case of existing perineural or lymphovascular invasion are used.

In palliative, inoperable situations or for symptomatic metastatic manifestations, hypofractionated dosage plans with single doses from 2.5–3.0 Gy up to a TD from 36–45 Gy are applicable (analogous to other tumor entities).

Use of special techniques (for example, brachytherapy) may be considered in individual situations, potentially with major therapeutic benefit. In view of the low evidence concerning this therapy, however, the decision should be made for each individual case during an interdisciplinary tumor board discussion.

### Drug therapy

*Chapter authors*: Ulrike Leiter, Alpaslan Tasdogan, Selma Ugurel

Given the rarity of these tumor entities, there is little evidence concerning the use of systemic therapies in inoperable or metastatic sweat gland carcinomas. Currently, chemotherapy is still the gold standard for metastatic tumors. Protocols of first‐line therapy include platin derivatives used in up to 60 % of the patients, often in combination with 5‐fluorouracil (5‐FU) or capecitabine, while cyclophosphamide, doxorubicin, or vincristine in combination with bleomycin represent second‐line therapies.^13^ A review by Mijamoto et al. on 28 patients receiving chemotherapy because of advanced eccrine porocarcinoma,^121^ showed an objective response rate of 31.3 %, suggesting a rather moderate to low chemosensitivity of these tumors. According to a recent systematic review and meta‐analysis by Abdullah et al. comprising the primary care of more than 1,000 patients with porocarcinoma, systemic therapy was only required for a minor fraction of < 1 % of the patients.^147^ In all cases, this consisted of chemotherapy in combination with either surgery or radiotherapy.

Targeted therapies like trastuzumab have been used for disease stabilization in tumors with HER2/neu expression.^13^ Individual case reports on the use of EGFR inhibitors, especially cetuximab, in metastatic sweat gland carcinoma exist.^7^, ^20^, ^148^, ^149^ In some cases this was used in combination with paclitaxel, resulting in one case in complete response of distant metastases over a follow‐up period of 6 months.^149^ A study by Denisova et al. showed that mutations in the *TP53* gene and genetic alterations in HRR‐associated genes (high HRD score) are common in porocarcinomas (in approximately 50 % of cases). Accordingly, PARP inhibitors may present a potentially promising option for targeted therapy.^6^

Immune checkpoint inhibition with PD‐1 antibodies represents another potential therapeutic option. PD‐L1 expression was identified in 13 % of sweat gland carcinomas.^16^ Individual cases with partial or complete response for 18 and 22 months, respectively, on second or subsequent therapies have been described.^150^, ^151^, ^152^, ^153^ Moreover, there is a case report showing partial remission for more than 12 months on first‐line treatment with pembrolizumab.^154^ A better response might be achieved in UV‐associated tumors.

In addition, intralesional therapeutic options may be considered. A case report described a complete remission of metastatic porocarcinoma after intralesional administration of interferon‐alpha plus interleukin‐2.^155^

Based on the currently available date, the chances of success of systemic therapy in sweat gland carcinoma are limited. Therefore, it seems useful to determine the tumor mutational burden in the tissue, the presence of a UV signature, the PD‐L1 expression in the tumor, and to search for driver mutations that may present a target for individualized targeted therapy.^6^

## FOLLOW‐UP

*Chapter author*: Ulrike Leiter

To date, there are only few studies on the follow‐up of sweat gland carcinomas in the literature. In addition to frequent recurrence, hidradenocarcinomas, porocarcinomas, digital papillary adenocarcinomas, and pilomatrix carcinomas also show a tendency to metastasis formation.^27^, ^156^, ^157^, ^158^ Given that more than 60 % of the patients with metastatic tumors develop both lymphogenic and primarily hematogenic recurrence within 2 years, follow‐up intervals between 3 and 6 months are recommended in the first two years after the initial diagnosis.^156^, ^159^ Subsequently, controls should be performed every 6 months or once a year until 5 years after the initial diagnosis, if possible.^133^

The follow‐up should include examination of the entire skin including palpation of lymph node stations and lymphatic drainage routes. Instruction for self‐examination is also advisable.^160^ Given that only few data concerning the follow‐up schedule in adnexal tumors are available, follow‐up according to the Mannheim scheme, modified according to the S3 guideline follow‐up scheme for patients with high‐risk cutaneous squamous cell carcinoma, is recommended (Table 7). Follow‐up intervals and the use of imaging techniques may be adapted in an individual and risk‐adapted manner. Moreover, instruction of patients for self‐examination is advisable. Regular ultrasound examinations are recommended, in particular, for the follow‐up of patients with enhanced risk of recurrence or metastasis.^20^, ^161^ Tomography by CT, PET‐CT, or MRI may be performed according to clinical indication and dependent on the disease stage. Given the rarity of these tumors and the potentially aggressive and lethal courses, access to a skin tumor center is desirable.

**TABLE 7:** Follow‐up schedule for sweat gland carcinomas (adapted from the Mannheim follow‐up scheme for malignant adnexal tumors of the skin and modified according to the S3 guideline follow‐up recommendations for patients with high‐risk cutaneous squamous cell carcinoma).^1^ ^6^ ^2^

	Clinical examination^*^		Sonography scar, in‐transit and regional lymph nodes		Tomography
	*Year 1–2*	*Year 3–5*	*Year 1–2*	*Year 3–5* ^***^	
Primary tumor	2 x	1 x	–	–	
Recurrent tumor	4 x	2 x	2 x	1 x	If metastases or advanced tumors are suspected
Tumors with locoregional, lymph node or distant metastases	4 x	4 x	2–4 x	1–2 x	Individually^**^ If metastases or advanced tumors are suspected

* History, examination of entire skin, inspection and palpation of lymph node stations and lymphatic drainage routes.

** If metastases or locally advanced tumors are suspected for assessment of deeper infiltration and if perineural growth is suspected.

*** Except for digital papillary adenocarcinoma where follow‐up is recommended for up to 10 years.

## CONSENSUS‐BUILDING PROCESS

The updated version of the guideline was created on behalf of the *Scientific Work Group on Dermatological Oncology of the German Cancer Society* and the *German Dermatological Society*.

The most important recommendations of this guideline are summarized in Table 8. Concerning the management of conflicts of interest: Experts with potential conflict of interest did not participate in the formulation of recommendations for relevant topics. Conflicts of interest of the individual experts were assessed by the guideline coordinators; the guideline coordinators were evaluated by the guideline representative of ADO/DKG.

**TABLE 8:** Summary of the most important statements and recommendations of the S1 guideline on sweat gland carcinomas (as of 2025).

Topic	Statement/recommendation according to guideline
Epidemiology	Very rare entities; < 0.05 % of all malignant skin tumors; After basal cell carcinoma most frequent group within adnexal carcinomas; Increasing incidence; With exception of few entities (for example, signet ring carcinoma of the skin), women and men are largely equally affected; First manifestation more often at an advanced age.
Etiopathogenesis	Development usually *de novo*, rarely arising from benign counterparts; Risk factors: ultraviolet radiation, radiotherapy, immunosuppression, human papillomaviruses, genetic aberrations, genetic syndromes (Brooke‐Spiegler syndrome), underlying hematoproliferative diseases
Classification	Subdivision into more than 20 entities; Classification into eccrine or apocrine carcinomas not always possible and irrelevant for prognosis and therapy; Clinical‐pathological TNM classification according to the 9th edition of the UICC classification, effective as of January 1, 2026; Most important entities: porocarcinoma, hidradenocarcinoma, malignant mixed tumor of the skin, spiradenocarcinoma / cylindrocarcinoma (frequent in Brooke‐Spiegler syndrome), syringocystadenocarcinoma papilliferum, extramammary Paget's carcinoma (be aware of pitfall inflammatory dermatosis), microcystic adnexal carcinoma (typically on cheeks), digital papillary adenocarcinoma (typically on fingers/toes), adenoid cystic carcinoma, mucinous carcinoma
Clinical appearance	Nonspecific clinical features. Excision usually on tentative diagnosis of benign cyst, basal cell carcinoma, squamous cell carcinoma; Mostly localized in head and neck area
Diagnosis	Excisional biopsy / excision for diagnostic confirmation; Histopathological criteria: apocrine or eccrine differentiation, cell atypies, nuclear pleomorphisms, crowding of nuclei, numerous mitoses, infiltrative growth; Immunohistochemical markers: a. o. cytokeratin (CK) 7, gross cystic disease fluid protein 15 (GCDFP‐15), carcinoembryonic antigen (CEA), epithelial membrane antigen (EMA), nuclear protein of testis (NUT); No established diagnostic criteria of dermoscopy or other non‐invasive in vivo diagnostics; Imaging in case of risk factors in primary staging and, if necessary, in follow‐up: locoregional sonography, computed tomography/magnetic resonance imaging to assess extension of primary tumor and exclude metastasis formation; Sentinel lymph node biopsy (SLNB): no general recommendations, case‐by‐case decision based on clinical and histopathological risk factors
Prognostic factors	Given the rarity, no specific staging exists; classification into the stages local disease, lymph node metastasis, and distant metastasis is useful; Metastasis initially occurs locally (*per continuitatem*), later to regional lymph nodes and distant organs (mostly lungs); Risk factors for local recurrence/metastasis: positive excision margins, large and thicker tumors, perineural invasion, high mitotic rate, lymphovascular invasion
Surgical therapy	The surgical approach should be defined for each individual case based on entity and localization; Treatment of choice: complete surgical excision with entire histopathological margin control (microscopically controlled surgery, MCS); Alternative: wide local excision (WLE) with safety margin of at least 1 cm, in microcystic adnexal carcinoma 2 cm (high local recurrence rate); In extramammary Paget's carcinoma recommendation for collecting preoperative mapping biopsies to determine tumor extension
Radiotherapy	No prospective studies; available data based on case series or retrospective monocentric observations; Recommendation for radiotherapy should be provided for each individual case in the interdisciplinary tumor board; Indications for radiotherapy: anticipated high morbidity due to radical tumor surgery, extensive / recurrent / multifocal tumor, residual tumor (R1/R2), lymph node metastases; In most tumors, postoperative adjuvant radiotherapy of the tumor bed including, if necessary, the regional lymph node station, is a case‐based interdisciplinary decision taking risk factors into account
Drug therapy	No prospective studies; available data based on case series or retrospective monocentric observations; Recommendation for drug therapy should be provided for each individual case in the interdisciplinary tumor board; Chemotherapy is the treatment of choice, combination therapy with platinum is recommended, response rate approximately 30 %; Targeted therapies possible, for example, with HER2 inhibitors (if expressed) or EGFR inhibitors (if overexpressed) in combination with chemotherapy, if appropriate; For immune checkpoint inhibitors (PD‐1), therapeutic response has been reported only in individual cases
Follow‐up	No systematic studies; Follow‐up care adapted to the follow‐up scheme for high‐risk cutaneous squamous cell carcinomas (S3 guideline) is recommended including results of clinical examination, sonography, and tomography, if appropriate

*Guideline coordinators*: Prof. Dr. med. Mirjana Ziemer, Leipzig; PD Dr. med. Michael Erdmann, Erlangen

*Representative of ADO/DKG*: Prof. Dr. med. Selma Ugurel, Bielefeld

## CONFLICT OF INTEREST STATEMENT

A.A., A.BA., D.H., C.EB., A.F., C.H., T.M., L.R., S.R., S.S., C.S. declare no conflicts of interest. F.M. has received travel support or/and speaker's fees or/and advisor's honoraria by Novartis, Roche, BMS, MSD, Pierre Fabre, Sanofi, Immunocore and Regeneron, outside the scope of the submitted work. J.U. has received honoraria or travel expenses from the following companies in connection with advisory board activities or speaking engagements: Amgen, BMS, GSK, Immunocore, Leo Pharma, MSD, Novartis, Pierre Fabre, Rheacell, Roche, and Sanofi, in each case outside the scope of the submitted work. S.U. declares research support from BMS, Merck; speakers honoraria from BMS, Merck, MSD, Regeneron; advisory board honoraria from BMS, Menarini Stemline, Merck, MSD, Regeneron; and meeting and travel support from Almirall, BMS, IGEA, MSD, Pierre Fabre, Sun Pharma, in each case outside the scope of the submitted work. M.Z. received reimbursement of travel expenses from Pierre Fabre and Sun Pharma as well as honoraria for lectures from Regeneron, Sanofi, Sun Pharma, Bristol Myers Squibb, AstraZeneca, Johnson & Johnson, and Delcath, outside the scope of the submitted work. J.C.B. reports funding from Alcedis, Bristol Myers Squibb, HTC Molecular Diagnostics, IQVIA, Merck Serono, and Regeneron as well as honoraria for advisory activities from Almirall Hermal, Amgen, Boehringer Ingelheim, ICON Clinical Research, Merck Serono, Pfizer, Regeneron, Sanofi, and Sun Pharma, in each case outside the scope of the submitted work. U.L. received reimbursement of travel expenses and/or honoraria for lectures and/or honoraria for advisory activities from Novartis, MSD, Pierre Fabre, Regeneron, Sanofi, Sun Pharma, and Almirall Hermal as well as funding from MSD, in each case outside the scope of the submitted work. C.W. received reimbursement of travel expenses and honoraria for lectures from AstraZeneca, Bristol Myers Squibb, Novartis, Pierre Fabre, Sanofi, Sun Pharma, and Takeda, in each case outside the scope of the submitted work. M.E. received honoraria for lectures and financial support for travel expenses and congress fees from Novartis, Pierre Fabre and Immunocore, in each case outside the scope of the submitted work.
